# Tolerance niche expansion and potential distribution prediction during Asian openbill bird range expansion

**DOI:** 10.1002/ece3.7456

**Published:** 2021-03-24

**Authors:** Yu Lei, Qiang Liu

**Affiliations:** ^1^ College of Wetlands Southwest Forestry University Kunming China; ^2^ National Plateau Wetlands Research Center Kunming China

**Keywords:** Asian openbill, ecological niche model, niche expansion, range expansion, tolerance niche

## Abstract

It is prevalent to use ecological niche models in the analysis of species expansion and niche changes. However, it is difficult to estimate the niche when alien species fail to establish in exotic areas. Here, we applied the tolerance niche concept, which means that niche of species can live and grow but preclude a species from establishing self‐sustaining populations, in such fail‐to‐establish events. Taking the rapidly expanded bird, Asian openbill (*Anastomus oscitans*), as a model species, we investigated niche dynamics and its potential effects on the population by Niche A and ecospat, predicted potential distribution by biomod2. Results showed that niche expansion has occurred in two non‐native populations caused by the tolerance of colder and wetter environments, and potential distribution mainly concentrated on equatorial islands. Our study suggested that the expanded niche belongs to tolerance niche concept according to the populations' dynamics and GPS tracking evidence. It is essential to consider source populations when we analyze the alien species. We recommended more consideration to the application of tolerance niche in alien species research, and there is still a need for standard measurement frameworks for analyzing the tolerance niche.

## Graphical abstract

A tropical bird, Asian openbill, has naturally spread to northward (such as China) and southward (such as Malay Peninsula) but not yet established. We illustrated our point on tolerance niche concept associated with Asian openbill expansion, then concluded that tolerance niche expansion occured both in two new distributed populations.

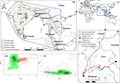

## 1 INTRODUCTION

Species geographic range is an expression of species niche in the environmental space (Sexton et al., 2009). Range shifts have already occurred in many species under climate change and globalization of human transport and trade networks (Parmesan & Yohe, 2003; Root et al., 2003; Seebens et al., 2017). Accordingly, researchers have shown an increased interest in untangling questions of ecological niche related to species distribution. Such as whether niche is conservatism in varied situation and scale (Peterson et al., 2011; Wiens & Graham, 2005), what is the relationship between niche breadth and range at latitudinal gradients (Boyle et al., 2020; Lancaster, 2016; Saupe et al., 2019; Sunday et al., 2011), how will organisms response to a novel environment (Davidson et al., 2011; Polechová et al., 2009; Spence & Tingley, 2020; Sutter & Kawecki, 2009; Szűcs et al., 2017). These critical scientific questions often derive from a story of organisms succeed or fail to establish when they arrived at a new area beyond their native range, because such alien species may pose challenges to the integrity of local ecosystems and biodiversity conservation (Tingley et al., 2014; Vilà et al., 2010). Therefore, it is necessary to study changing ecological niches and distribution trends when a species range expands rapidly.

Over the past two decades, ecological niche models (ENM) have been widely implemented in biological invasion and macroecology research, such as measuring niche characteristics and comparing differences between native and novel niches, analyzing habitat suitability, and predicting future spatial distribution trends (Beukema et al., 2018; Broennimann & Guisan, 2008; Mandle et al., 2010; Ørsted & Ørsted, 2019; Parravicini et al., 2015; Tingley et al., 2014; Villaverde et al., 2017). Studies of niche dynamics under climate change, which have received more attention, show that the biological invasion often accompanies niche shifts (Broennimann et al., 2007; Gallagher et al., 2010; Stiels et al., 2015). However, such conclusions can be suspicious because of insufficient evidence or inappropriate modelling frameworks, for instance, lack of analyses in environmental space, and excluding factors like partial niche filling, sampling bias, or the unequal availability of environmental conditions (Guisan et al., 2014; Peterson et al., 2011; Petitpierre et al., 2012; Qiao et al., 2017). Hence, an appropriate modelling method should be chosen rather than blind trust (Joppa et al., 2013; Qiao et al., 2015).

Besides methodology problems, nearly all relevant studies to date have tended to focus on established (also termed invasive, introduced, or naturalized) species rather than non‐established ones. It means former study objects can persist and exist indefinitely in their environmental space, which should be suggested to the fundamental niche or realized niche concept (Hutchinson, 1957). A conceptual confusion to researchers is that realized niche used to be a subset of fundamental niche (Peterson et al., 2011) but sometimes it can be larger than the fundamental niche in a source‐sink situation (Pulliam, 2000; Soberón, 2007). That existence of species may include a situation that individuals can survive only, fail to establish, or have not yet established. Sax et al. (2013) developed the niche components and proposed a marginal zone outside of the fundamental niche, a “tolerance niche” area where individuals of a species can live and grow but preclude a species from establishing self‐sustaining populations. Tolerance niche clarifies the ambiguous terminology in some specific cases between fundamental niche and realized niche, also prevalent among many species, especially great dispersal abilities species. Here, we select a representative species with natural range expansion to investigate its niche dynamics and the potential effects on the population. More importantly, we try to understand whether there is a tolerance niche in niche shift cases.

The Asian openbill (*Anastomus oscitans*) is a large tropical wading bird that specializes in forage molluscs. It belongs to the stork family Ciconiidae and is mainly distributed in South Asia and Southeast Asia, including India, Sri Lanka, Bangladesh, Myanmar, Thailand, and other countries (Elliott et al., 2020). Observed data in recent years describe a large‐scale phenomenon of Asian openbill expansion: the first records in China in 2006 (Wang, 2007), Malay peninsula in 2008 (Lim et al., 2008), and Singapore in 2013 (Low et al., 2013). The range of expansion can be divided into two directions: south to southern Thailand, Malaysia, Singapore and north to northern Vietnam and China, and the number of individuals has increased from a few to thousands each year (Han et al., 2016; Jiang, 2010; Liu et al., 2015; Low et al., 2013). Data showed that no breeding behavior was found in any population of Asian openbill in the new distribution areas over 10 years (Han et al., 2016; Low et al., 2013; Zainul‐Abidin et al., 2017), and presented that the majority of individuals are subadult birds, and the population is more in summer and less in winter in China (Han et al., 2016; Lei et al., 2017). We refer to the niche where the storks occurred in expansion ranges as tolerance niche and where the storks occurred in all ranges as realized niche in our study. To date, less is known about those individuals' source population and the niche changes to which extent when a tropical bird stays throughout years both in the lower and higher latitudinal new range. Due to abundant food resources and lack of biotic competitions in expansion areas (Lei et al., 2017), we prefer to investigate how storks respond to various abiotic conditions during range expansion. Here, we will illustrate our point on tolerance niche concept associated with Asian openbill expansion, the following questions were addressed: (a) did the niche change during the range expansion? (b) if niche changed, how to understand the difference between niches of native and exotic ranges? And (c) based on the present niche characteristics, how might the Asian openbill spread in the future?

## 2 METHOD

### 2.1 Occurrence data

We divided all species records into five subsets: all occurrences, present, native, north, and south population data. In detail, all occurrence data on the Asian openbill consist of Internet sources and field surveys. Data were downloaded from the Global Biodiversity Information Facility (GBIF, www.gbif.org), which contained 26,925 original distribution records from 1970 to 2020. Field data consisted of 43 records obtained from our surveys in southwest China in 2016 and 2017. Then, 26,968 records were obtained by combining the above two datasets as all occurrences of Asian openbill (Figure 4a).

**FIGURE 1:**
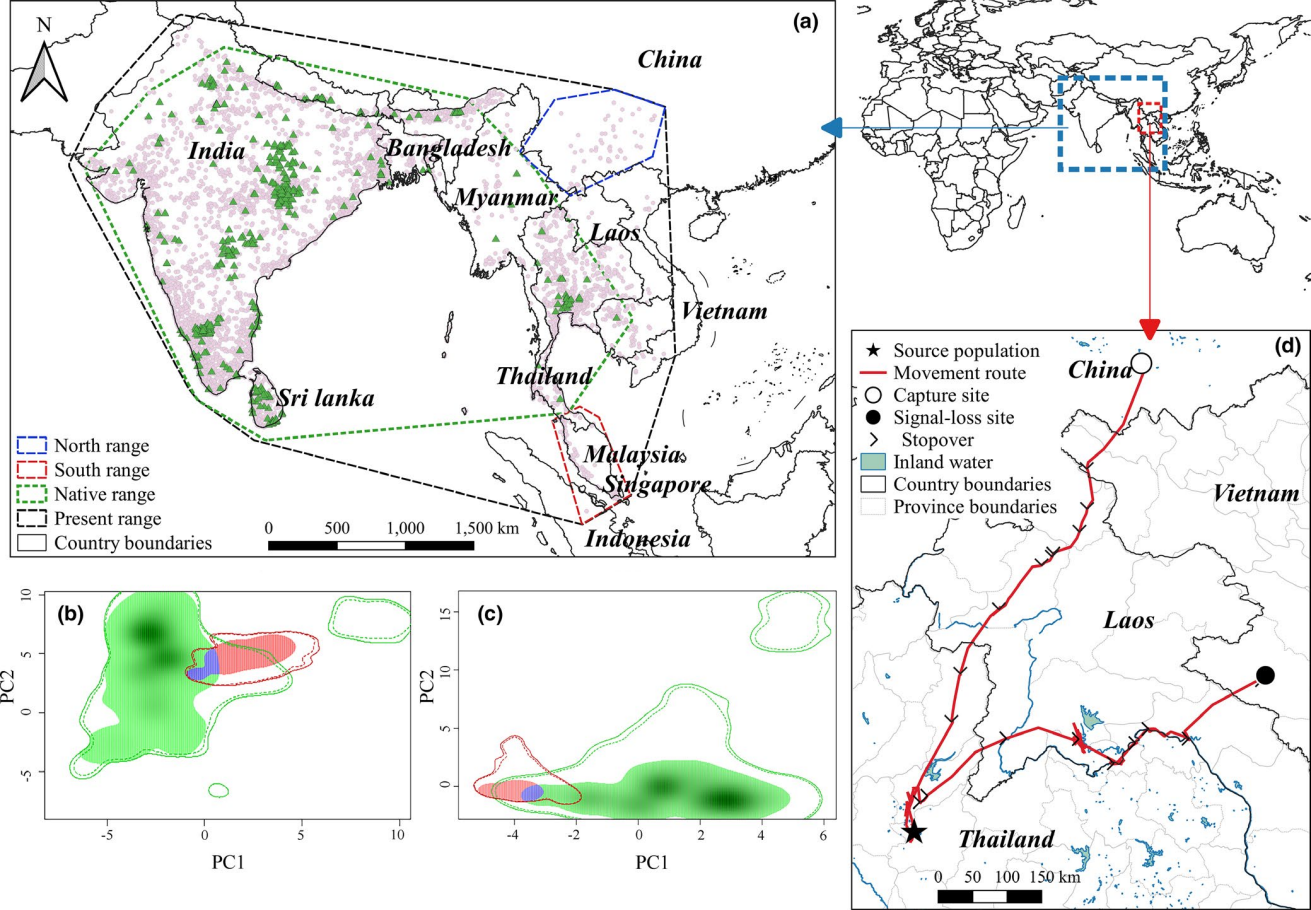
Study ranges and population data in our niche analysis (a). Green triangles represent the native population. Pink points represent thinned data which include new north population within the blue polygon and new south population within the red polygon (a). Panels b and c plot of niche overlap by species in native range (green) and new range (red). Panel b compares the native population with north population; panel c compares the native population with the south population. Purple areas represent the overlapped niche among populations. Panel d shows that an Asian openbill in China can fly back to Thailand

Sampling bias has pervasive adverse effects on the results of niche modelling (Peterson et al., 2011). To minimize sampling bias in geography space, we removed obvious erroneous data, such as duplicates, poor precision records, and conspicuously inaccurate data. Some transient and occasional records were also removed to eliminate individual interference as we focus realized niches on the seasonal or year‐round population of different ranges. Considering the relative independence of the data, we kept only one occurrence point for each 5 km. All the processes were implemented using the Wallace package in R version 3.5.1 (Kass et al., 2018), and the data finally reduced to 3,980 records as the present population (Figure 1a).

Documentations revealed that range expansion of the storks is a continuous process that may be induced by population explosion since 1997 in Chao Phraya River, Thailand (Han et al., 2016). So we extracted data before 1997 from the present data as the native population (*n* = 514). Little is known about expansion processes in Thailand and Vietnam, so we extracted the Chinese data as the north population (*n* = 41), and records in Malaysia, Singapore, and Indonesia represented the south population (*n* = 12) (Figure 1a). Here, the data in the north and south populations also exclude some transient and occasional records. All the occurrences that fall outside the native range we displayed in Figure 4a.

### 2.2 Environmental variables and study area

We used 19 bioclimatic variables with a resolution of 2.5 min obtained from WORLDCLIM as predictors (www.worldclim.org). To reduce the underlying analysis error caused by multi‐collinearity environmental variables both in environmental space and geographical space, we selected the principal component analysis (PCA) to transform those 19 variables into uncorrelated linear combinations for subsequence analysis. As a result, we used the first two principal components for ecospat analysis, three principal components for Niche A and biomod2 analysis, which summarized 71.9% and 82.3% of the environmental data's overall variation, respectively, to represent environmental variability across the landscape for model analysis. PC1 was mainly composed of quarterly precipitation factors and annual average temperatures, PC2 mainly represented temperature variability and annual precipitation, and PC3 was related to minimum and average monthly temperature (see Appendix S1).

The inappropriate extent of study area may lead to many problems in ENM (El‐Gabbas & Dormann, 2018). Considering the species distribution ranges and movement abilities, we delimited six study areas in our analysis, including the native, north, south, present, calibration, and projection ranges. The first three ranges are used in ecological niche analysis by ecospat and Niche A. The background context of Niche A was generated from the present range. We made a global model based on calibration ranges then transferred to projection ranges in biomod2. More specifically, the native, north, south, present, calibration ranges were based on the five occurrence subsets we termed above by MCP with 1‐degree buffer, which covers all environments that have been accessible to the species in the study periods. Ranges of native, north, south, and present were generated from the occurrence subsets with corresponding names (Figure 1a). The calibration range was set up from all species occurrences which covered all species accessible ranges so far (Figure 4a). The projection range contained Sino‐Japanese, Oriental, Oceanian and Australian zoogeographic regions proposed by Holt (2013) (Figure 4a). We provided such extensive projection range because Asian openbill has shown an ability to cross Andaman sea, plateau, and mountains. Hence, we infer the bird able to arrive at suitable conditions within our projection range.

### 2.3 Ecological niche analysis

**FIGURE 2:**
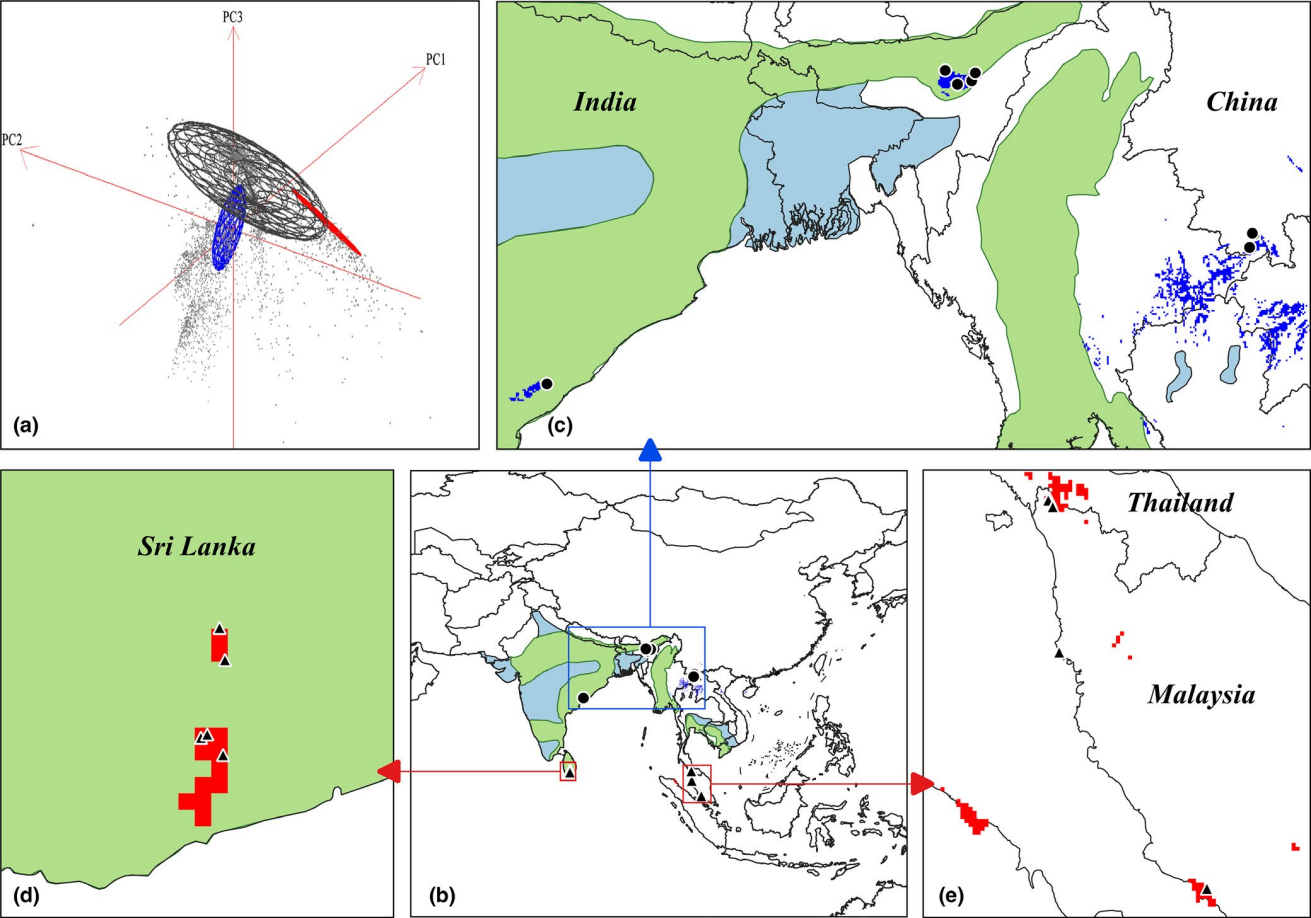
Realized niche overlap among the native population (black), north population (blue), and south population (red) in Niche A (a). We extracted the overlapped part in ecological space projected to geographic space as analogous environments, then added occurrence points within those environments and species distribution map to infer populations' residential tendency in similar climatic conditions (b). Green area indicates the sedentary range and blue area indicates wintering range. Panel c shows that two points in China occupied analogous environments (blue range) with five points within the native sedentary range. Four records within south population (e) utilized similar climatic conditions (red range in e and d) with Sri Lanka's sedentary populations (d). Species distribution map from Birdlife Internation (http://datazone.birdlife.org)

Using occurrences to compare the differences in environmental attributes of recorded sites between the native and exotic ranges in environmental space is a main approach for quantifying niche changes (Guisan et al., 2014). Ecospat and Niche A were used to identify niche overlap and climatic condition utilization of each population. We used the first three components in Niche A software (Qiao et al., 2016) to display minimum volume ellipsoid (MVE) generated from native, north, and south populations. First, we determined analogous environments from overlapped niche and then projected these environments in geographical space to identify which individuals utilized those analogous conditions. Finally, according to species distribution map, we inferred the new distribution population's potential residential status (Elliott et al., 2020) (Figure 2).

Principal component analysis is an efficient way to quantify the niche overlap and minimize sampling bias (Broennimann et al., 2012), it is also an essential procedure for ecospat package in our niche analysis (Di Cola et al., 2017). The first two PCA axes were selected at a resolution of 100 × 100 grids to compare species density differences in the native, north, and south populations. Meanwhile, we used the Schoener's *D* metric to calculate the degree of overlap and examine niche conservatism, including four indices overlap (*D*), unfilling, stability, and expansion in ecospat (Guisan et al., 2014; Petitpierre et al., 2012; Warren et al., 2008). When we overlapped the native and exotic niches, the proportion of exotic niche that did not overlap with the native niche was termed expansion, the exotic niche overlapping with the native niche was termed stability, and the native niche that did not overlap with the exotic niche was termed unfilling (Guisan et al., 2014). Overlap (*D*) measures niche overall match between two entities, from 0 (niche have no overlap) to 1 (niche identical) (Warren et al., 2008). In order to test whether or not the niche is equivalent and similar, we used equivalency and similarity tests in ecospat (Broennimann et al., 2012; Warren et al., 2008). Niche equivalency tests were performed to determine whether the native and exotic niche generated by occurrences were identical, and sample data were randomly run 1,000 times to calculate the overlap scores and actual overlap. When the actual overlap value was within 95% of the simulated value, the niche equivalency hypothesis could not be rejected. Niche similarity assesses whether the niches of two ranges are higher or lower than the random expectation generated when the niche of one study area overlaps with the background of another study area after 1,000 randomizations. We hypothesize the north and south population niche is more different from the native population niche. So we set the alternative parameter as “lower” in ecospat to test the niche divergence. If the *p*‐value smaller than 0.05, it means the niche overlap is greater than difference random expectations. Finally, we identify which climatic variables of new distribution populations had exceeded the native population in terms of occurrence density values when the expansion occurred in niche analysis.

### 2.4 Potential geographic distribution

Lots of ENMs or species distribution models can predict potential spatial distributions of species. Although there are many model options, no single optimal metric is widely applicable in this field (Qiao et al., 2015). Besides, the emerging consensus algorithm could balance the performance of multiple models (Marmion et al., 2009), but results are ambiguous compared to a single model (Breiner et al., 2015; Zhu & Peterson, 2017). Hence, we used the four individual models – generalized linear model (GLM), random forests (RF), generalized boosted model (GBM), and maximum entropy (Maxent) – and an ensemble model to predict species distribution in the biomod2 package (Thuiller et al., 2009).

First, we combined all occurrence data and three principal components generated a calibration model within the calibration range, then the potential distribution was predicted after transferring the calibration model to the projection range. Specifically, 70% of occurrence data were used for model training and 30% for model testing. Second, we selected the partial receiver operating characteristic (PROC) as the model evaluation criteria; in contrast with the AUC method, PROC eliminates the misleading effects of pseudo‐absence data on the model application and emphasizes the crucial role of the omission rate in model evaluation (Anderson et al., 2003; Peterson, 2006). An AUC ratio of 1 implies that the niche model is no better than a random prediction, and a larger AUC ratio indicates better discrimination in the partial ROC approach (Peterson et al., 2008). Then, we chose a model with the highest PROC value for potential distribution analysis. Given the criticism of the complexity and transferability of Maxent default settings, we chose the minimum delta AICc and the maximum average AUC test values in the ENM eval package as optimizing parameters for Maxent (refer Appendix S3) (Muscarella et al., 2014). Besides, due to the potential extrapolation uncertainty of the model in the transfer, we still used ExDet to determine the novel environmental parameter (Type 1 novelty) (Figure 4) (Mesgaran et al., 2014).

Finally, to compare whether the probability of two new populations was higher than other potential areas within the Asian openbill accessible area, we randomly created 1,000 occurrence sites in the projected range. A probability value of 0–1 was generated in the optimal model with the highest PROC value to compare species' occurrence probability in the native, north, south, and random population.

### 2.5 Satellite telemetry

Although Yang et al. (2019) provided direct evidence that Thailand's bird can fly into Mengzi, China, we still lack more information to identify the species' source population in new ranges. Here, we assume that Mengzi's bird can fly back to Thailand due to the seasonal fluctuated population dynamics in China. In July 2018, we rescued a subadult Asian openbill trapped by fishing nets at Changqiao lake (130°22′21″E, 23°25′20″N) in Mengzi City, Yunnan Province, China. After ensuring the physical health, we equipped the stork with Yuehai^®^ GPS‐GSM logger (25 g, 1.7% of body mass, 65 × 25 × 29 mm). We scheduled transmitters to fix positions every 1–4 hr and chose the accuracy of GPS points within 10 m in data analysis.

## 3 RESULTS

### 3.1 Ecology niche analysis

Niche analysis in ecospat revealed that the realized niche to the north and south populations only partially overlapped with the native population niche (Stability = 14.30%, 28.80%, respectively) and more than 70% exceeded the native population niche (Figure 1b,c, Table 1). Schoener's *D* quantification results showed that compared to the native population, niche divergence of the north population was significantly higher than expected (*p* < 0.05), representing significant niche expansion (Table 1). Moreover, there was incomplete niche match in overlap *D* value (the maximum value was 0.05) (Table 1), indicating that in recent years, the Asian openbill niche changed or resulted in an unbalanced regional distribution.

**TABLE 1:** Niche overlap results comparing two non‐native population with the native population

New population	Expansion (%)	Stability (%)	Unfilling (%)	Niche overlap (D)	Niche equivalency (P)	Niche similarity (P)
North	85.70	14.30	56.11	0.02	0.03*	0.66
South	71.19	28.81	76.87	0.05	0.67	0.73

Note

The niche equivalency and similarity tests are the selected “lower” option to emphasize niche divergence. *p* < 0.05 indicates that niches are more divergent or different than expected by chance. Asterisk denotes statistical significant.

**FIGURE 3:**
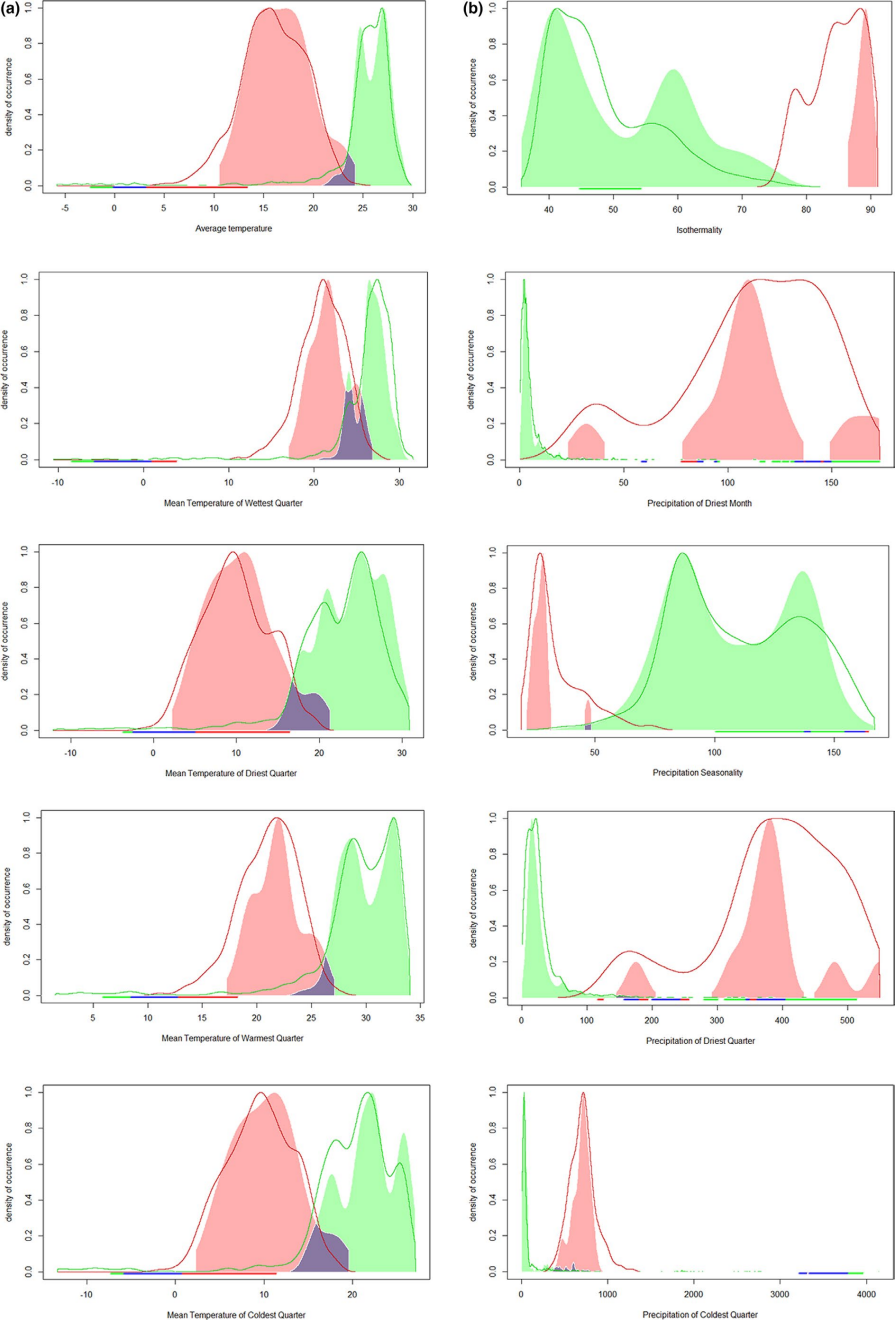
Plot of the occurrence density values of some environment variables among populations. Panel a represents the native (green) compared to the north population (red). Panel b represents the native (green) compared to the south population (red). The colored areas represent occurrence density curve, the lines represent marginal response curve. Purple is the area where two populations' occupancy range overlapped. We found lower temperature within the north population and greater precipitation within the south population than native's

Comparing the occurrence density values of each environment variable among three populations, we found lower temperature within the north population and greater precipitation within the south population than native's (Figure 3, refer detail in Appendix S2.1, S2.2). The results indicated that the north population occupied colder environments, and the south population occupied wetter environments than the native population. The border niche occupancy expanded their realized niche.

Analyzing analogous environments utilized situation from overlapped niche component in Niche A, we found the two records within north population occupied analogous environments with a native sedentary population in India (Figure 2c). Four records within south population utilized similar climatic conditions with Sri Lanka's sedentary populations (Figure 2d,e).

### 3.2 Potential spatial distribution

**FIGURE 4:**
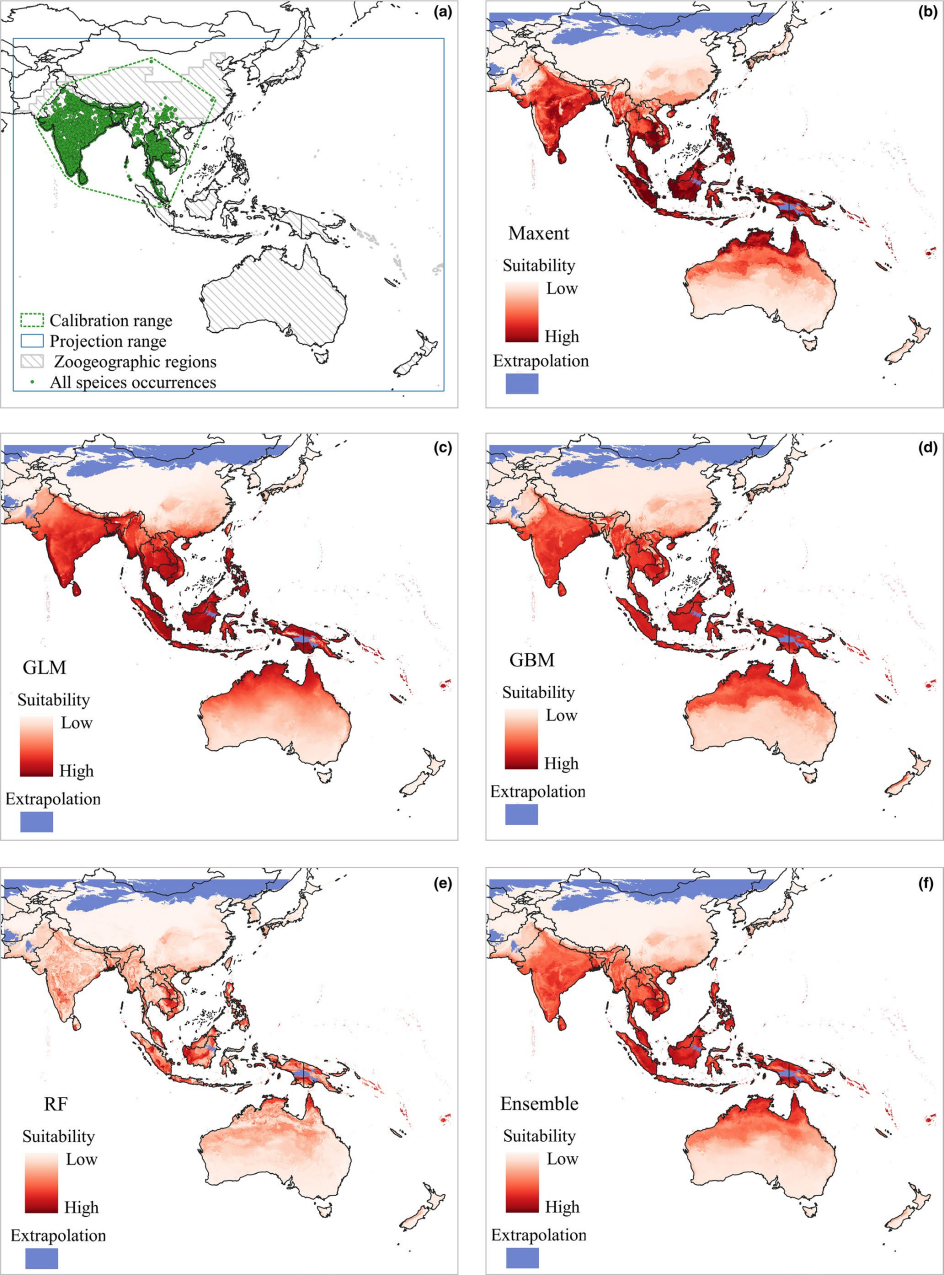
Study ranges we used in spatial analysis (a). Calibration range delimited by all occurrences and projection range delimited by zoogeographic range (a). Using four individual models and an ensemble algorithm predicted potential distribution of Asian openbill, including Maxent (b), GLM(c), GBM(d), RF (e) and ensemble model (f). Blue areas indicate regions with novel climate conditions (extrapolation) with respect to those characterizing the projected range

**FIGURE 5:**
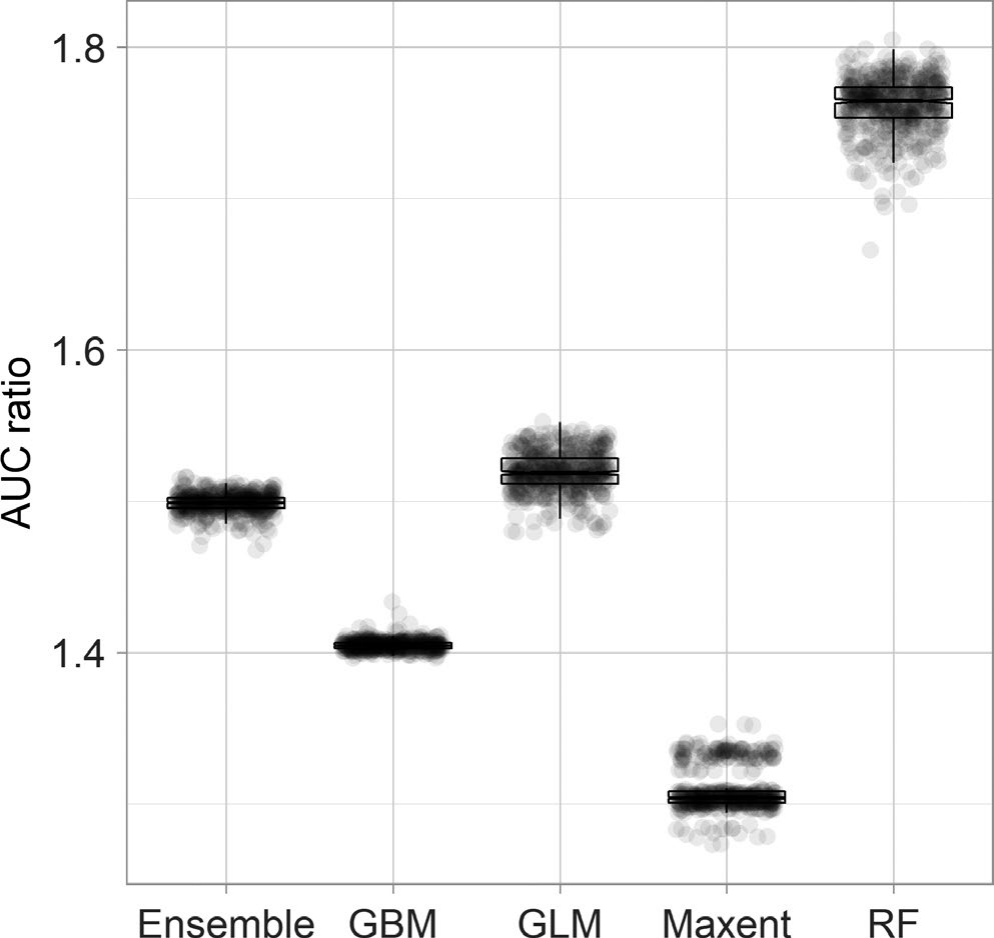
Evaluation of five models using partial ROC ratios. Boxplots denote AUC ratios for 500 replicates using 50% of evaluation occurrences in each replicate and a 5% omission error

The suitability prediction map of four individual models and the ensemble model showed that suitable value of species was negatively correlated with latitude gradient (Figure 4). Out of the native range, several islands near the equator were more suitable for the Asian openbill. North Australia also had high suitability values, but some high latitudes in the East Asia regions, such as China, Mongolia, Japan, and the Korean Peninsula, were less suitable. However, a few sub‐suitable areas still occurred in south China. Based on the PROC evaluation, we found that the AUC ratio value of the RF model was significantly higher than other models, so it was chosen for probability analysis (Figure 5).

**FIGURE 6:**
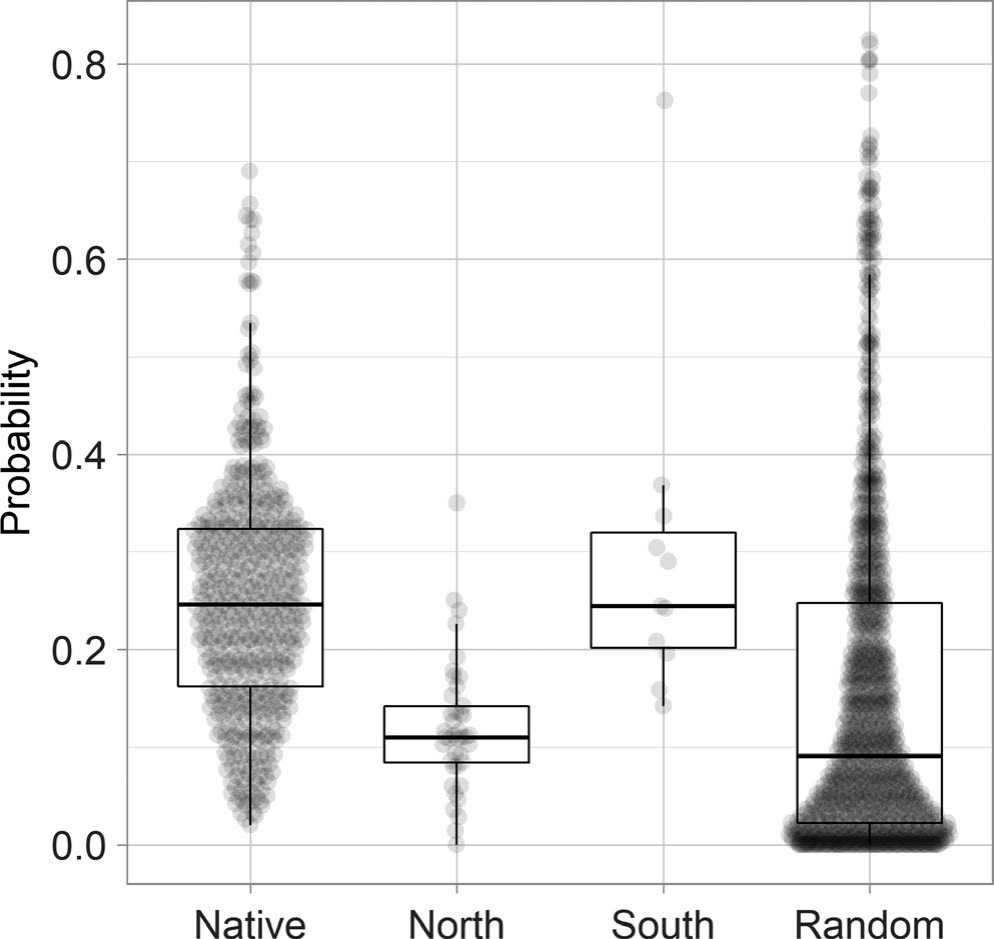
Probability of occurrence at four population sites

The occurrence probabilities of the native, north, south, and random populations were extracted from the RF model. We found that the south population's median value is close to native (Native, Median = 0.25, South, Median = 0.24) and the north population similar to random (North, Median = 0.11, Random, Median = 0.09) (Figure 6). The south population had high potential distribution probability while the north population was almost randomly distributed.

### 3.3 Satellite track

We obtained a partial annual circle movement route and received a total of 2,112 GPS positions in 225 days (Figure 1d). The tracked stork left Mengzi City, China, on August 29, 2018, and arrived at the Changwat Uttaradit in Thailand after 18 days. The linear distance between the two sites is 736 km, and cumulative route distance is 907 km. After 90 days in Thailand, the bird set out a northeastward move across Laos until lost the signal in Vietnam on April 8, 2019. Associated with recovered records from Bueng Boraphet, Thailand to Mengzi, China (see detail in Yang et al., 2019), we obtained the information that there may have a flyway between Thailand and China. The source population of the north range at least partially from Thailand.

## 4 DISCUSSION

### 4.1 Tolerance niche expansion during range expansion

The occurrence data used in this study were composed of breeding and non‐breeding populations, so our study populations' niches were collectively referred to as the realized niche. Compared with the native realized niche, the north and south populations showed niche expansion. As opposed to typical niche expansion, the Asian openbill distributed in China and the Malay Peninsula has no breeding records over 10 years (Han et al., 2016; Lei et al., 2017; Zainul‐Abidin et al., 2017). It means the expanded niche is beyond the bounds of the fundamental niche. In this specific case, as we mentioned in the introduction, it is necessary to classify which niche components can hold sustainable populations and which cannot. Pulliam (2000) proposed a source‐sink dynamics theory to describe that topic. In this theory, species living in source habitats can maintain their local reproduction exceeds local mortality, and sink habitats are reverse. Due to the no breeding records and significant seasonal fluctuated population size (Han et al., 2016; Lei et al., 2017; Zainul‐Abidin et al., 2017), we speculate that this pattern of population change should belong to the source‐sink dynamics. Our satellite track study and the previous banded Asian openbill records (Yang et al., 2019) in China provided pieces of evidence on the role of the north range as a sink habitat for the species. However, the source‐sink theory cannot include all cases that reproduction smaller than mortality in ecosystem. The tolerance niche, which developed from source‐sink dynamics, adaption, and range edges theories, contains that range conditions in which individuals can only survive and lack the ability to establish self‐sustaining populations (Sax et al., 2013). Moreover, Sax suggests that the tolerance niche is a sophisticated systemic concept like the fundamental niche, also affected by dispersal limitations, species interactions, and other factors. Therefore, we preferred to define the expended niche in our study as the tolerance niche.

Our measurements represented two distinct niche expansion characters. The north population had a stronger tolerance to low temperatures while the south population tended to occur in more humid areas. Indeed, field surveys have found that some individuals in China can stand the temperature below zero Celsius (Han et al., 2016; Lei et al., 2017). The expanded tolerance niche represented a more complete niche breadth for Asian openbill. Niche breadth has a close relationship with range size (Kambach et al., 2019). As a tropical species, Asian openbill is able to endure a wide range of environmental conditions like other endotherms (Strubbe et al., 2015), it is a reason we select all occurrences to build global model in spatial analysis as the native ranges may underestimate climate tolerance of species (Bocsi et al., 2016).

In this study, we also try to discuss the consequences of tolerance niche to species. According to utilization in analogous environments among populations, we inferred birds from new ranges who lived in analogous conditions possible to reproduce based on the species distribution map because sedentary birds were identified in the native range under similar conditions (Figure 2). Here, we noticed that the similar environmental conditions both belong to the fundamental niche and tolerance niche because we defined the niche concept based on whether reproduction occurred in study ranges. That involves a more complicated question about what makes a species establish successfully. Many factors should be considered as propagule pressure (Lockwood et al., 2005), conspecific density (Taylor & Hastings, 2005), and species life‐history traits (Blackburn et al., 2009). However, we discuss the reproduction possibility only based on climatic conditions and lack of references on that non‐overlapped niche in this study. For reference, there have been many birds that have spread to low and high latitudes at the same time and successfully established, such as the cattle egret (Lovette & Fitzpatrick, 2016), the ring‐necked parakeet (Strubbe et al., 2015), the Egyptian goose, and the Common waxbill (Blackburn et al., 2009). We still lack enough data to predict reproduction in new ranges, but we should mention that tolerance niche in our study may turn into fundamental niche.

As far as we know, this is the first time that the concept of tolerance niche has been applied to birds. We believe that using this concept can help us understand species establishment mechanisms and has an important consequence in species conservation (Sax et al., 2013). However, many questions need to be untangled for the concept application in the future, that is, Are the tolerance niche has a distinct boundary with fundamental niche? What makes tolerance niche become fundamental niche (or can understand as when sinks become sources in source‐sink theory (Lavigne et al., 2020))? Which factors can preclude a species establishing self‐sustaining populations in animals? Moreover, there is a need for a standard methodology framework to quantitatively identify the tolerance niche.

### 4.2 The potential distribution of Asian openbill

We found that species potential distribution was correlated with latitude gradient. The high suitable area of the Asian openbill we predicted was constrained to near equator area, especially some islands. The unsuitable range mainly distributed at high latitude. All the models showed that there are some specific patches of sub‐suitable area in south China. Considering the excellent dispersal ability of Asian openbill, the bird is possible to expand to those areas. However, many factors affect species existence, especially for the rapid demographic changing species. Here, we focus not only on the suitable map of model but also on understanding the expansion pattern of Asian openbill.

Occurrence data and our suitable map revealed that Asian openbill has continuously distributed pattern with high density at the center and gradually decline toward the range edge, but some individuals in the new range edge are fragmentally distributed (Figure 4a). The difference between the central population and the edge population may be caused by widely varying biogeographic region, trophic group, body size (Gaston & Gaston, 2003), and life‐history stage (Burton et al., 2010). Also, edge populations are assumed to have greater variability in survival and reproduction (Sexton et al., 2009). Thus, demographic on edge population is suggested more fluctuant (Elliott & Evenden, 2012). So the results of occurrence probability analysis are acceptable, which showed that the south and native populations have a greater probability of appearing in the species range, whereas the north population seems randomly distributed (Figure 6).

The previous study on tracking flyways of Asian openbill in Thailand reported that some individuals moved between south and central Thailand (Ratanakorn et al., 2018), even fly to northeast India and Bangladesh during the non‐breeding season (McClure, 1998). It could be argued that the “over‐migration” occurred in Thailand when the storks initially migrated north, bringing a few migrants to locations out of their regular migratory range as vagrants (Ralph & Wolfe, 2018), and arriving in southwest China. Based on our niche and distribution analysis results, perhaps the north populations are far from the niche centroid then accelerated the dispersal rate (Ingenloff et al., 2017), resulting in a wide distribution range extremely unstable in the early expansion stage. In conclusion, we predicted the equatorial islands are more suitable as potential distribution ranges while the north populations will still fluctuate in the future.

## CONFLICT OF INTEREST

The authors declare they have no competing interest.

## AUTHOR CONTRIBUTIONS

**Yu Lei:** Conceptualization (equal); Data curation (equal); Formal analysis (lead); Investigation (equal); Methodology (lead); Resources (lead); Software (lead); Validation (equal); Visualization (lead); Writing‐original draft (lead); Writing‐review & editing (lead). **Qiang Liu:** Conceptualization (equal); Data curation (equal); Funding acquisition (lead); Investigation (equal); Project administration (lead); Resources (supporting); Supervision (lead); Validation (equal); Writing‐original draft (supporting); Writing‐review & editing (supporting).

## Supporting information

Appendix S1‐S3

## Data Availability

Species location data available at https://www.gbif.org. Bioclimatic variables available at https://www.worldclim.org. The processed occurrence data of this study are available from the Dryad Digital Repository (https://doi.org/10.5061/dryad.83bk3j9qz). The PCA and bioclimatic variables analysis results and Maxent parameter optimisation results uploaded as Appendix S1–S3.
