# Supplementary material for: Tolerance niche expansion and potential distribution prediction during Asian openbill bird range expansion
Source: Ecol Evol. 2021 Mar 24;11(10):5562–74. doi: 10.1002/ece3.7456 (PMC8131807; doi:10.1002/ece3.7456)
Supplement: Supplementary file 1 — Appendix S1‐S3 [file ECE3-11-5562-s001.doc]

Tolerance niche expansion and potential distribution prediction during Asian openbill bird range expansion

Supporting information

**Appendix S1.** Environment Variables in model analysis used and contribution in three axes

| **Variable** | **Description** | **Unit** | **PC1** | **PC2** | **PC3** |
| --- | --- | --- | --- | --- | --- |
| bio1 | Annual Mean Temperature | ℃ | 0.288881162 | 0.189299915 | 0.090616425 |
| bio2 | Mean Diurnal Range | ℃ | 0.261908192 | 0.24090143 | 0.097735338 |
| bio3 | Isothermality | % | 0.298869628 | 0.145299509 | 0.09346053 |
| bio4 | Temperature Seasonality | % | 0.254726275 | -0.192116286 | -0.297222073 |
| bio5 | Max Temperature of Warmest Month | ℃ | 0.225595829 | -0.031816754 | -0.429904272 |
| bio6 | Min Temperature of Coldest Month | ℃ | 0.127553925 | -0.374966501 | 0.286476018 |
| bio7 | Temperature Annual Range | ℃ | -0.039080385 | 0.40421843 | -0.178608834 |
| bio8 | Mean Temperature of Wettest Quarter | ℃ | 0.231721257 | -0.051903602 | -0.433753089 |
| bio9 | Mean Temperature of Driest Quarter | ℃ | 0.140872376 | -0.374195375 | 0.268520262 |
| bio10 | Mean Temperature of Warmest Quarter | ℃ | 0.136830989 | -0.21357592 | -0.417622689 |
| bio11 | Mean Temperature of Coldest Quarter | ℃ | 0.1366336 | -0.27964878 | 0.136870503 |
| bio12 | Annual Precipitation | mm | -0.185706283 | 0.241185434 | 0.140199818 |
| bio13 | Precipitation of Wettest Month | mm | 0.186541438 | -0.120526457 | 0.257220587 |
| bio14 | Precipitation of Driest Month | mm | -0.26744765 | 0.028826584 | -0.067138816 |
| bio15 | Precipitation Seasonality | % | 0.239204842 | 0.294867783 | 0.128686985 |
| bio16 | Precipitation of Wettest Quarter | mm | 0.304356194 | 0.102039593 | 0.088392412 |
| bio17 | Precipitation of Driest Quarter | mm | -0.265951517 | 0.165726898 | -0.006928221 |
| bio18 | Precipitation of Warmest Quarter | mm | 0.265263457 | 0.217238092 | 0.064498561 |
| bio19 | Precipitation of Coldest Quarter | mm | 0.286987665 | 0.176146351 | 0.118653053 |

Appendix S2.1 Comparison of the occurrence density values of each environment variable between native and north population.


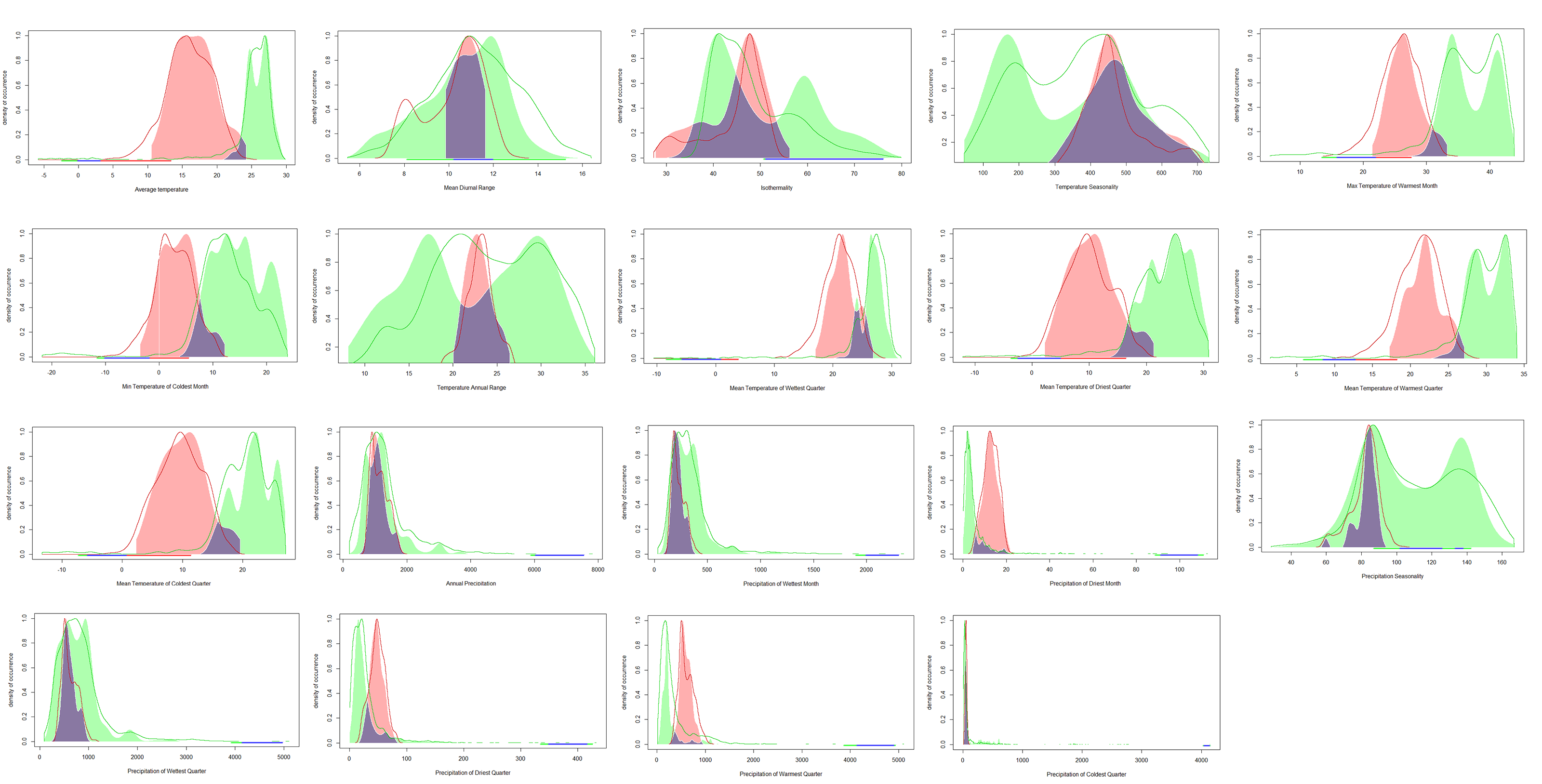


**Figure S2.1** Plot of the occurrence density values of all environment variables between native (green) and north population (red).

Appendix S2.2 Comparison of the occurrence density values of each environment variable between native and south population.


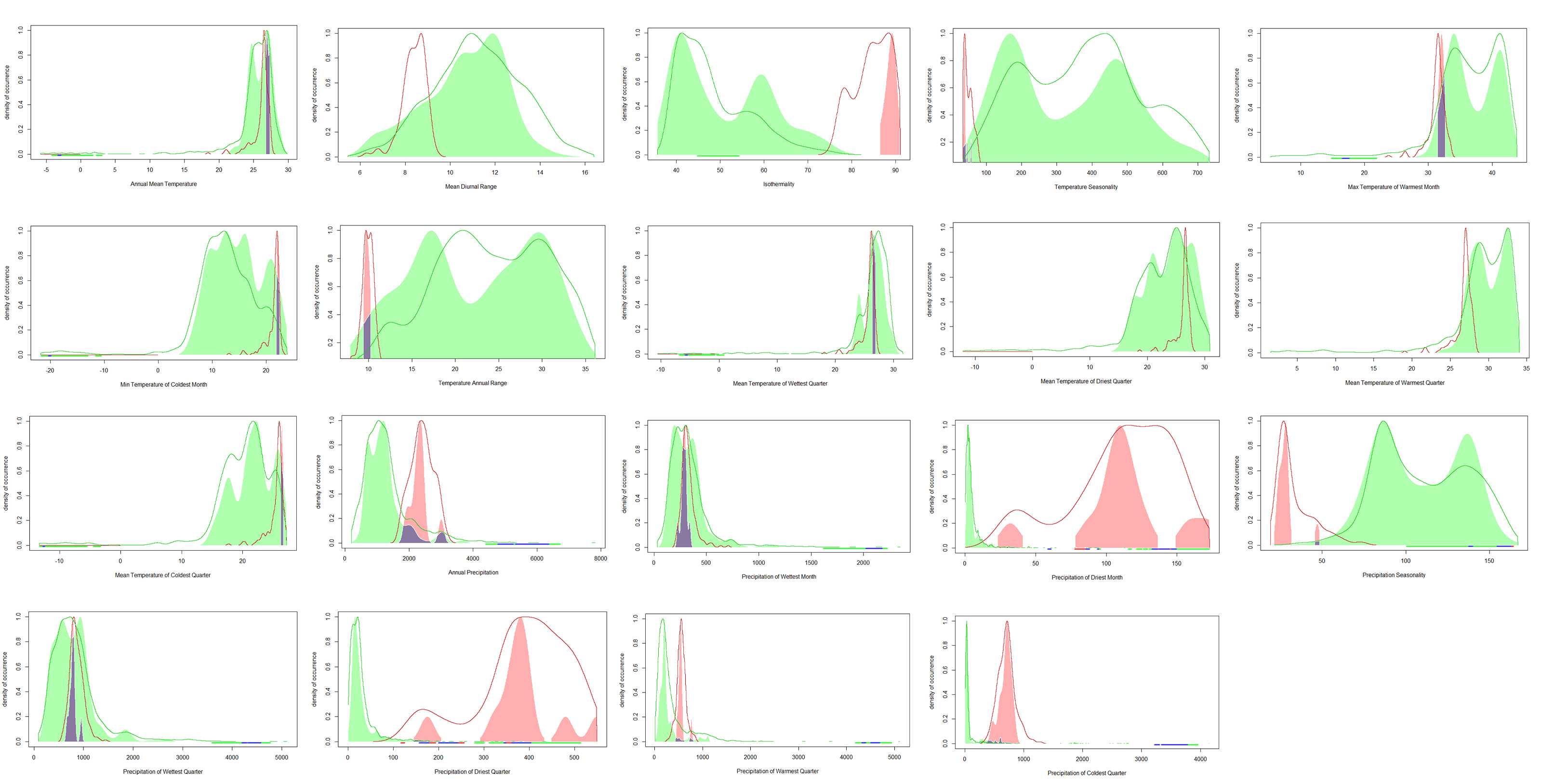


**Figure S2.2** Plot of the occurrence density values of all environment variables between native (green) and south population (red).

**Appendix S3. Maxent default parameter optimization.**


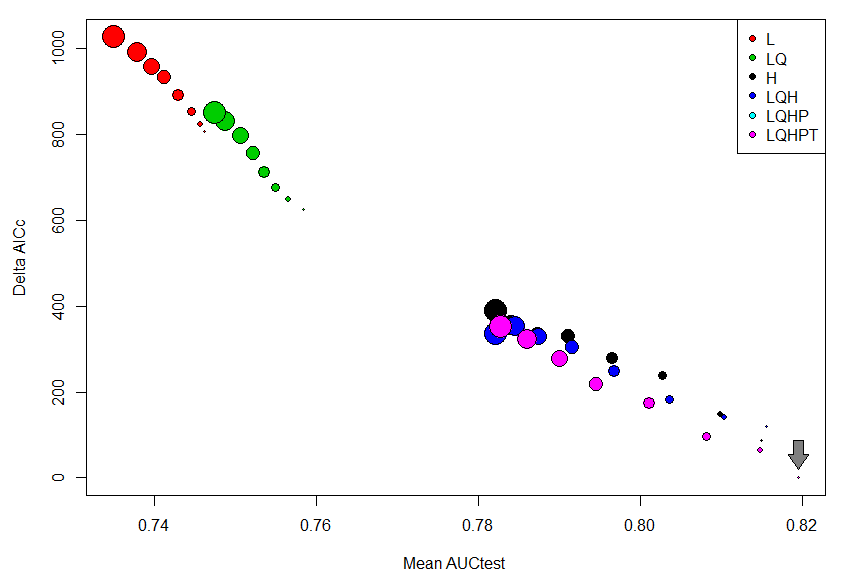
**Figure S3.** Adjust the regularization multiplier (RM) and feature combination (FC) values in the ENMeval package. The minimum value of delta AICc (vertical axis) and the maximum value of Average AUCtest (horizontal axis) are selected as the optimal parameter combination. The size of the circle represents the size of the RM value, and we chose the parameter combination of RM=0.5 and LQHPT indicated by the grey arrow.
